# Anesthetic Considerations for and Management of a Parturient With Jarcho-Levin Syndrome: A Case Report of Continuous Spinal Anesthesia for Preterm Cesarean Section

**DOI:** 10.7759/cureus.90303

**Published:** 2025-08-17

**Authors:** Ámbar Ramírez-Rodríguez, Julián J Zayas-Vélez, Dimaris Domínguez, Evelyn Carrero, Maria J Crespo

**Affiliations:** 1 Anesthesiology, University of Puerto Rico, School of Medicine, San Juan, PRI

**Keywords:** cesarean section, continuous spinal anesthesia, difficult airway, jarcho-levin syndrome, neuraxial anesthesia

## Abstract

Jarcho-Levin syndrome (JLS) is a rare congenital disorder marked by multiple vertebral abnormalities, a shortened spine, and rib fusion at the costovertebral junction. In addition to the typical physiological changes associated with pregnancy, patients with JLS face complications affecting their lungs, heart, spine, and mobility. Difficult airway management, pulmonary disease, and spinal deformities observed in these patients require a collaborative, multidisciplinary approach and meticulous perioperative planning. The presence of a challenging airway complicates the choice between general and neuraxial anesthesia. We present a case of a parturient with JLS who successfully underwent preterm cesarean delivery with continuous spinal anesthesia (CSA). After consultation with the multidisciplinary team, which included the Cardiology, Otolaryngology, and Obstetrics services, neuraxial anesthesia via a continuous spinal approach was chosen.

## Introduction

Jarcho-Levin syndrome (JLS) is a rare inherited genetic disorder characterized by a combination of short stature, vertebral malformations, and rib anomalies [1]. Often used as an umbrella term, JLS encompasses conditions such as spondylocostal dysplasia and spondylothoracic dysplasia, which result in a range of congenital defects affecting the spine and ribs. Associated deformities include scoliosis, a short neck, a shortened torso, a "fan-like" or "crab-like" rib cage, and a protruding abdomen [2]. The severity of JLS varies depending on the extent of skeletal malformations, particularly those influencing thoracic deformities, which can compromise cardiac and respiratory functions. JLS is also associated with multiple congenital cardiac anomalies, such as ventricular septal defects, atrial septal defects, and heterotaxy syndromes. Although rare, JLS is more prevalent in Puerto Rico, with an estimated one in 8,500 babies born with the condition each year [1]. Due to the rarity of JLS, coupled with the infrequent incidence of pregnancy among affected individuals, there is scarce literature on anesthetic management for parturients with this syndrome [3].

This case report highlights the anesthetic approach in a parturient with JLS who underwent a successful cesarean delivery using continuous spinal anesthesia (CSA), a significantly underutilized approach in clinical practice [4]. CSA was preferred over a single-shot technique because a full-term pregnancy increases the risk of a high spinal block with a single injection. CSA allows titration in small incremental doses to achieve the desired block level while minimizing the risk of high spinal complications, particularly in a patient with short stature and a potentially difficult airway. To our knowledge, this is the first reported case of CSA used in a patient with JLS.

## Case presentation

**Figure 1:**
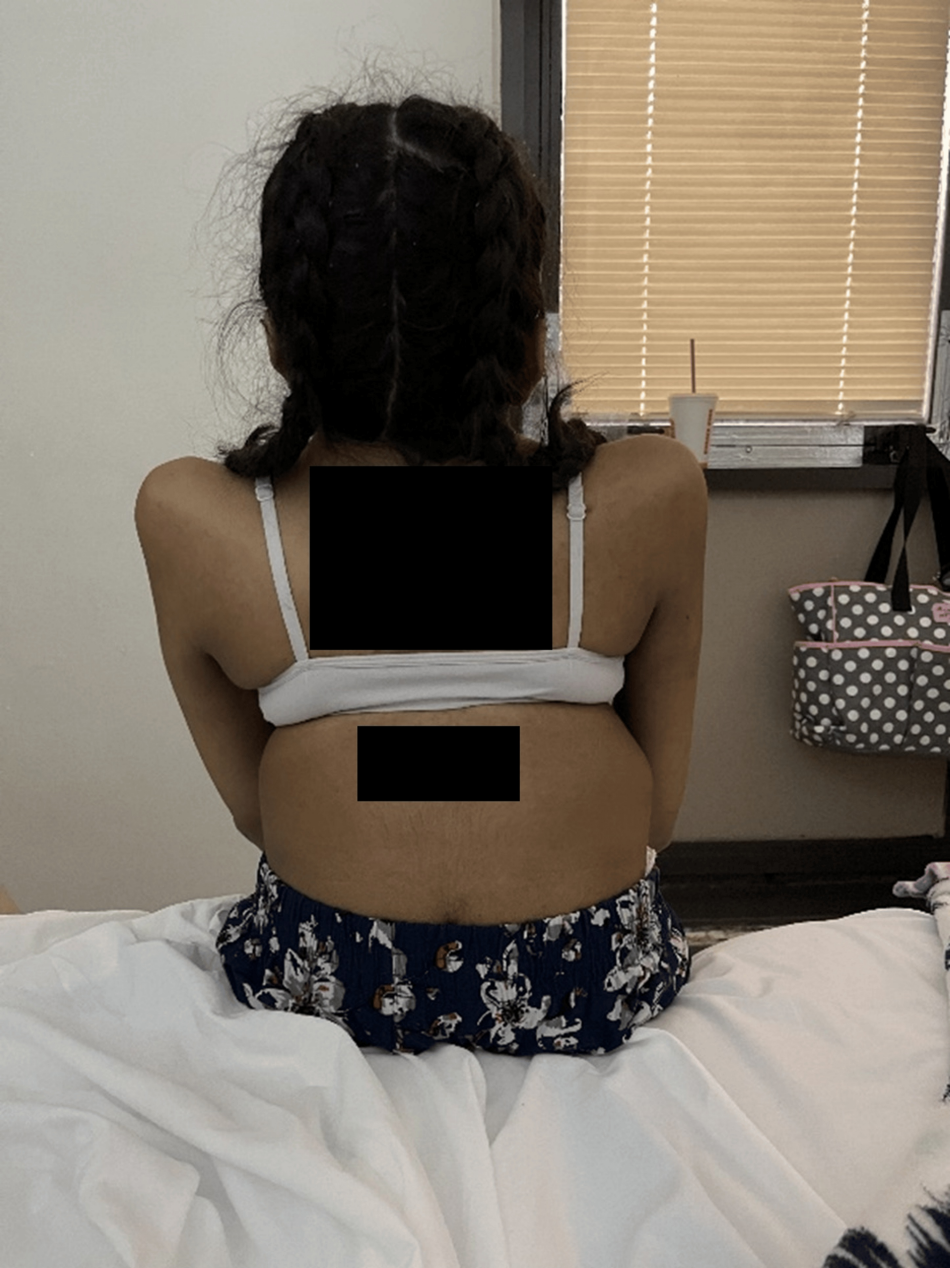
Posterior view of the patient seated upright in bed The image shows thoracic and lumbar spinal deformities, including rib cage asymmetry and scoliosis

**Figure 2:**
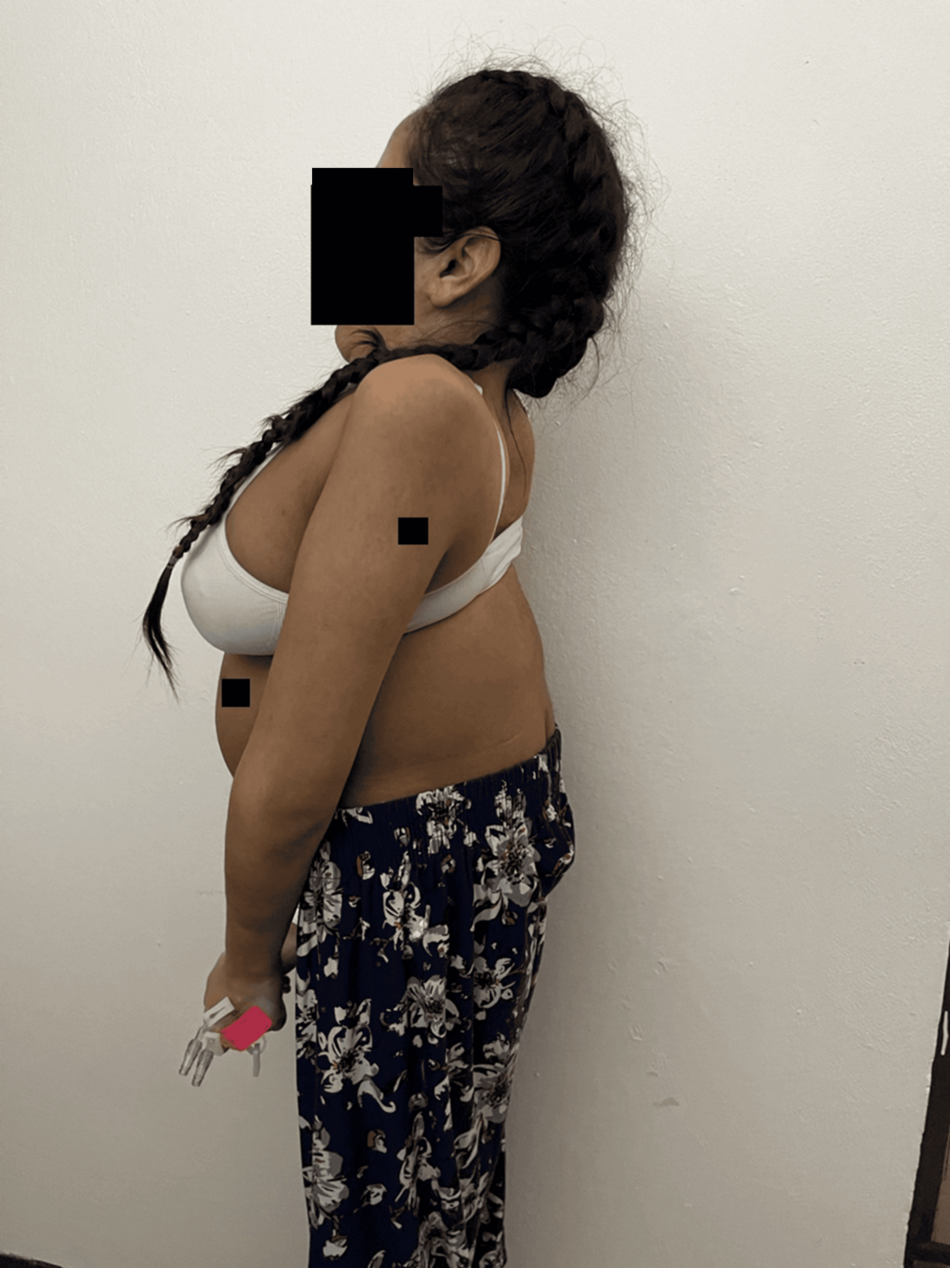
Standing lateral view of the patient The image shows thoracic kyphosis, forward head posture, and shortened trunk proportions

**Figure 3:**
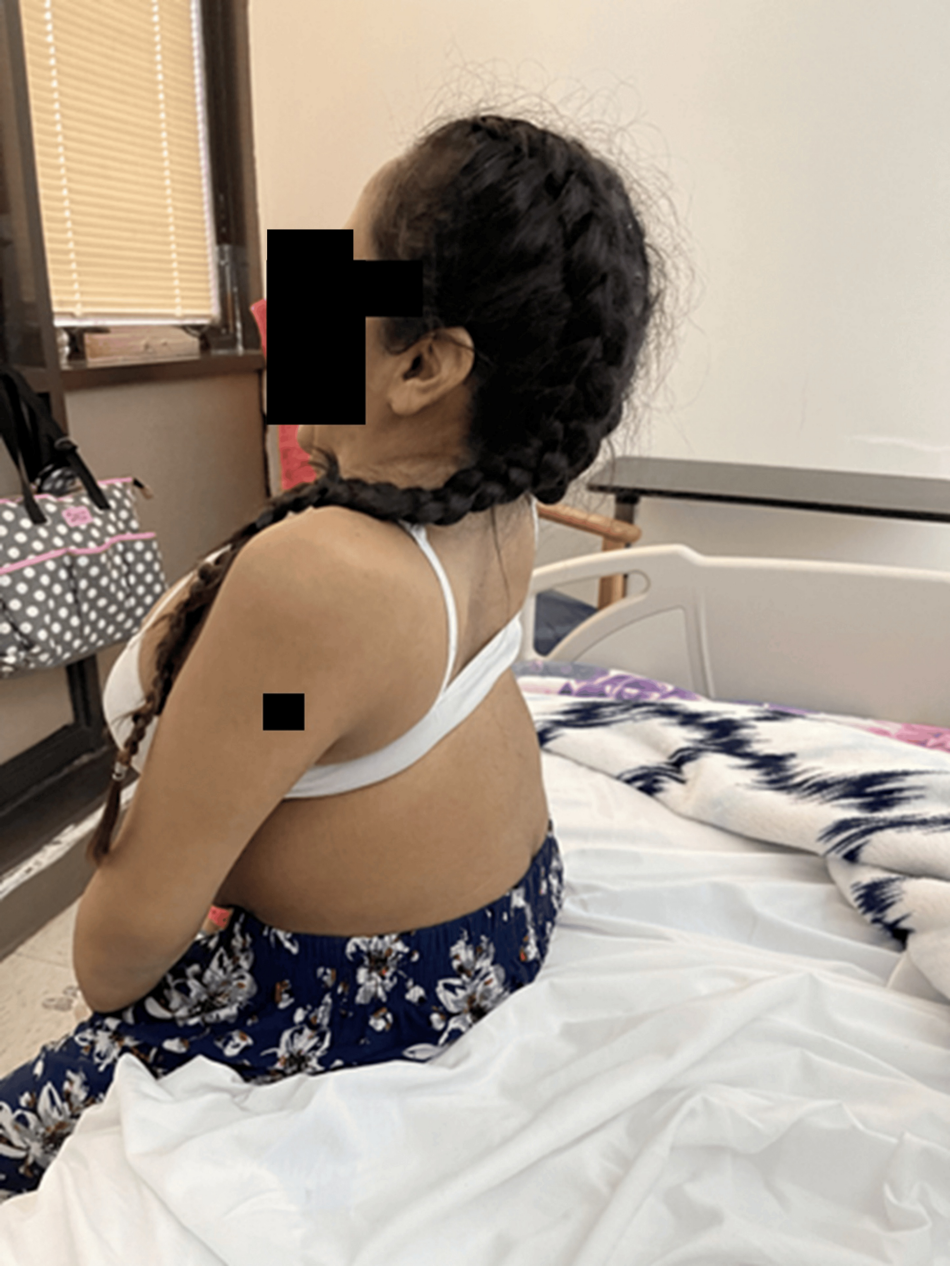
Lateral view of the patient seated upright The image shows pronounced kyphoscoliosis, reduced anteroposterior chest diameter, and prominent curvature of the spine. The scapulae appear elevated, and the thoracic contour is significantly altered

A 29-year-old Puerto Rican female, G1P0, at 30 weeks of gestation with JLS, arrived at the Obstetrics Emergency Room with complaints of shortness of breath and fatigue when walking. Physical examination findings were notable for short stature, a fixed neck, and scoliosis (Figures 1-3). She was 52 inches tall and weighed 35 kg at the time of assessment. The anesthesiology service was consulted to discuss and evaluate the anesthetic management in preparation for a possible emergent cesarean delivery.

**Figure 4:**
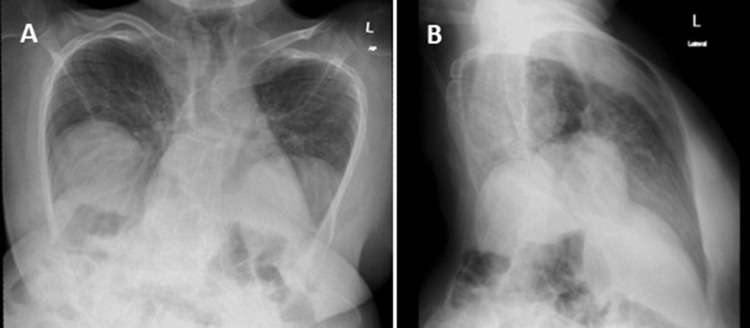
X-ray findings (A) Frontal anteroposterior, and (B) left lateral chest radiographs demonstrate fan-like rib orientation and abnormal thoracic contour, findings consistent with severe congenital spinal dysplasia, including pronounced thoracic lordosis and scoliosis

The patient's airway examination revealed no neck extension, limited mouth opening, and a Mallampati Class III, all factors that increased the likelihood of a difficult airway during the procedure. She denied any cardiac or respiratory complications related to her JLS diagnosis. Immediate preoperative vitals were as follows: heart rate (HR): 83 bpm, SpO₂: 96%, and BP: 110/76 mmHg. Pulmonary function tests were not ordered, as the patient denied any history of pulmonary complications. Radiographic imaging, including chest and upper spine radiographs, confirmed the clinical findings of a difficult airway (Figures 4A-4B).

**Figure 5:**
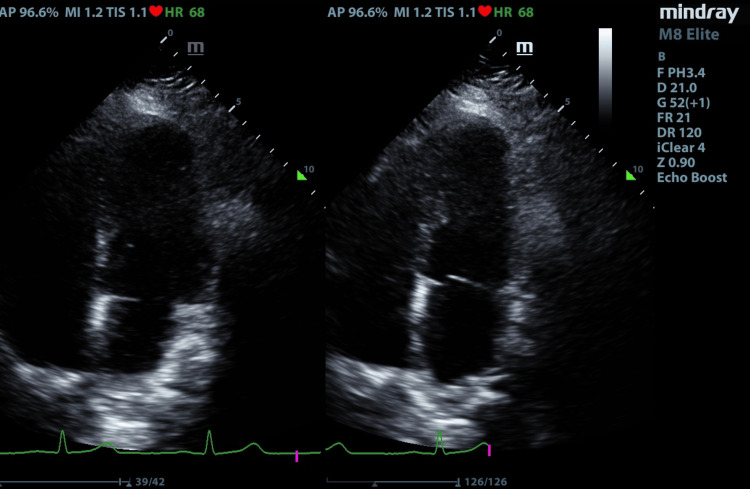
Two-dimensional transthoracic echocardiogram

Consultations were made with several specialties: cardiology for an evaluation with a transthoracic echocardiogram (Figure 5, Table 1), otolaryngology to assess the potential need for a surgical airway, and obstetrics to address concerns about abdominal retraction for proper visualization of the suprapubic area. During this visit, the patient was diagnosed with Mycoplasma pneumonia and admitted to the Obstetrics unit due to potential complications related to her JLS. She received appropriate antibiotic treatment, which successfully resolved her shortness of breath and fatigue. Following the resolution of her primary symptoms, a multidisciplinary team - including a maternal-fetal specialist, anesthesiologist, otolaryngology team, and neonatologist - evaluated her case. Based on their assessment, they recommended delaying delivery and scheduling an elective cesarean section at 34 weeks of gestation, provided the patient could tolerate it. The patient expressed understanding and gave verbal consent to the proposed plan.

**Table 1:** Results of two-dimensional transthoracic echocardiographic evaluation EF: ejection fraction; IVC: inferior vena cava; LVH: left ventricular hypertrophy; RAP: right atrial pressure; RV: right ventricle; RVSP: right ventricular systolic pressure; TAPSE: tricuspid annular-plane systolic excursion

2D transthoracic echocardiogram	
Rhythm	
Sinus	
Chambers	
Left ventricle	The left ventricle appears normal in size. Normal systolic function, EF 60-65%
	No LVH
	Normal diastolic function
	No wall motion abnormalities
Right ventricle	RV is normal in size
	RV function is normal by S' TAPSE
Left atrium	The left atrium is normal in size
	No clots noted
Right atrium	The right atrium is normal in size
	No clots noted
Valves	
Aortic valve	The aortic valve is structurally normal, unable to determine if trileaflet
	There is no stenosis on Doppler
	There is no regurgitation on color Doppler
Mitral valve	The mitral valve seems structurally normal
	No mitral regurgitation
	No mitral stenosis
Tricuspid valve	The tricuspid valve is structurally normal
	No tricuspid regurgitation
	No tricuspid stenosis
	RVSP <35
Pulmonic valve	The pulmonic valve is structurally normal
	No pulmonic valve regurgitation
	No pulmonic valve stenosis
Mass or thrombus	
No intracardiac mass or thrombus noted	
Vessels	
IVC normal and collapses >50% with inspiration, which suggests RAP of 3 mmHg	
Pericardium	
No pericardial effusion noted	

The patient's surgical history included a hip surgery performed three years ago at another institution, during which spinal anesthesia was successfully administered as the primary anesthetic. No intraoperative or postoperative complications were reported during that procedure. At 32 weeks of gestation, she experienced worsening shortness of breath and fatigue with ambulation and required multiple pillows for sleeping. Given her difficult airway and prior successful use of neuraxial anesthesia despite vertebral deformities, this anesthetic technique was preferred to minimize the risk of airway compromise and the potential need for surgical airway intervention. Following a discussion with the patient and the medical team, the anesthesiology team decided to proceed with the cesarean section under neuraxial anesthesia. The otolaryngology team was on standby to perform an emergency surgical airway if necessary, and difficult airway equipment was checked and made readily available. She received preoperative aspiration prophylaxis with sodium citrate (3 grams in 30 mL, PO) and metoclopramide (10 mg, IV). Upon arrival in the operating room, standard monitoring was initiated, including non-invasive BP (NIBP), pulse oximetry, and electrocardiography. Additionally, two intravenous lines were established: a 22-G cannula in the left hand and an 18-G cannula in the right hand.

The patient was placed in a sitting position for CSA placement. The palpation of the lumbar area identified a suitable intervertebral space around L3-L4, where local anesthetic was infiltrated. A 17-G Hustead needle was used to perform the lumbar puncture, and an epidural catheter (Arrow, Teleflex, Wayne, PA; catheter gauge: 19 G; depth at skin: 7 cm) was inserted into the intrathecal space. An initial dose of hyperbaric bupivacaine (1 mL, 0.75%) was administered through the catheter. The sensory blockade assessment revealed lateralization, with the right side (T8) greater than the left side (T12). After repositioning the patient and adjusting the intrathecal catheter, a repeat bolus of hyperbaric bupivacaine (1 mL, 0.75%) was administered. The final sensory blockade assessment remained sidelined, with the right side at T4 and the left side at T8. After consulting with the obstetric surgical team about the anesthetic level, the procedure began following the administration of supplemental local anesthetic infiltration with lidocaine (2%, subcutaneous) at the surgical site and adjunct sedation with ketamine (25 mg, IV) and midazolam (1 mg, IV). Surgical access to the uterus was challenging due to the displacement of the patient’s visceral organs by her abnormal thoracic structure. The procedure lasted one hour, with multiple anesthetic agents administered via a spinal catheter, IV lines, and local infiltration at the surgical site. The estimated blood loss was 600 mL.

A baby girl was delivered with Apgar scores of 8 and 9 at one and five minutes, respectively, and was transferred to the Neonatal ICU due to preterm birth. Following delivery, an oxytocin infusion was initiated without affecting blood pressure or heart rate. She remained hemodynamically stable throughout the procedure, with BP ranging from 140/90 to 110/55 mmHg and HR from 118 to 85 bpm. Intraoperatively, the patient received 2,250 mL of IV fluids, with a total operating room time of two hours and 15 minutes. For postoperative pain management, fentanyl (25 µg, IV) was administered via the intrathecal catheter, which was subsequently removed without complications. The patient was monitored in the Obstetrics Post-Anesthesia Care Unit for three hours before being transferred to the obstetrics ward. Postoperatively, the patient received a single dose of morphine (4 mg, IV) before transitioning to oral medications for continued pain management. She remained hospitalized for five days and was then discharged home in stable condition, with no signs or symptoms of post-dural puncture headache.

## Discussion

We discussed the successful anesthetic management of a 29-year-old primigravid woman with JLS who underwent cesarean delivery under CSA. Anesthetic management in these patients presents significant challenges due to multi-systemic anomalies, which can increase the difficulty of airway management and intravascular volume control. These factors heighten the risks associated with both general and neuraxial anesthesia, requiring an individualized approach. In this case, by administering neuraxial anesthesia via a continuous spinal approach, safe perioperative care was ensured. A thorough evaluation of our patient's unique anatomical and physiological considerations guided the development of a customized anesthetic plan, which resulted in a positive outcome. Patients with JLS have an altered spinal anatomy, increased risk of hemodynamic instability, and often present with a difficult airway, compromised pulmonary reserve, reduced circulating blood volume, and, in some cases, right heart failure secondary to pulmonary hypertension [5]. Therefore, when selecting the anesthetic approach for our patient, we carefully weighed these factors and evaluated the available options such as general endotracheal anesthesia (GETA), single-shot spinal (SSS) anesthesia, epidural analgesia (EA), and CSA.

While we selected CSA for our parturient patient with JLS, Bellamy et al. [6] used GETA for a JLS patient with severe spinal deformities undergoing cesarean delivery. Their decision was based on concerns about the uncertain location of the conus medullaris, the presence of significant kyphoscoliosis, and the possible presence of dural or subarachnoid scarring from prior corrective spinal surgeries. In our patient, however, these factors were not the primary concerns; instead, our decision to use CSA was guided by the risks of hemodynamic instability, difficult airway management, and significant blood loss. Despite concerns regarding the potential for post-dural puncture headache and cauda equina syndrome [7], CSA offers several advantages over GETA, including reduced intraoperative blood loss, faster maternal recovery, and superior postoperative analgesia. Nonetheless, in the current case, IV fluids and 1 liter of packed RBC were administered during surgery to maintain an adequate intravascular volume. Additionally, general anesthesia has been linked to higher rates of maternal mortality, cardiac arrest, difficult or failed intubation, hypoxemia, pulmonary aspiration, and intraoperative awareness compared to neuraxial techniques [8].

Among the available neuraxial options, SSS anesthesia was also considered. SSS offers several advantages, including lower local anesthetic requirements, technical simplicity, and the ability to achieve a dense motor block [7,9]. However, we selected CSA for its more predictable duration and improved hemodynamic stability through titratable dosing, which significantly reduces the risk of hypotension compared to SSS. Continuous spinal approach also provides a faster onset of sensory block, a shorter time to achieve surgical anesthesia, and more precise control of cephalad spread. In addition, CSA facilitates postoperative analgesia and may enhance maternal recovery. These benefits made CSA the most appropriate choice in this context. Recent literature supports the use of intrathecal opioids as adjuvants to spinal anesthesia in cesarean deliveries, including prolonged sensory blockade, improved postoperative pain control, and a reduced need for systemic opioids [8,10]. The combination of local anesthetics with intrathecal opioids allows for lower doses of each agent, thereby decreasing the likelihood of hypotension and dense motor block. Although this novel approach may have improved analgesia while better preserving cardiovascular stability in our high-risk patient, the supporting report was not published until recently and was therefore unavailable at the time of surgery.

EA, another neuraxial technique, was excluded due to its slower onset and higher rate of incomplete blockade. Achieving adequate sensory and motor block with EA often requires repositioning the patient, making it less suitable for cesarean delivery. Another concern in this patient was the large volume of local anesthetic needed for EA, which could increase the risk of cardiovascular instability from sympathetic chain blockade [9]. Although direct comparisons between CSA and EA are limited, some studies have compared CSA with continuous epidural analgesia (CEA), finding that CSA offers a faster onset, more effective sensory and motor blockade, and shorter recovery. CSA has also been linked to greater hemodynamic stability, with CEA showing a more significant drop in mean arterial pressure [11]. While data comparing CSA and EA in obstetric patients remains limited, the choice of technique depends on clinical context and patient-specific factors.

In patients with JLS, downward displacement of the abdominal organs makes surgical exposure of the uterus more challenging during fetal delivery. In our case, these anatomical differences, along with elevated intra-abdominal pressure, required adjustments to the standard cesarean technique. To improve access and facilitate delivery, a vertical supraumbilical incision was chosen instead of the traditional Pfannenstiel incision. Although effective, this approach led to a longer surgical duration, emphasizing the need for an anesthetic plan that sustained hemodynamic stability throughout the procedure. In anticipation of considerable blood loss secondary to a higher associated risk of blood loss [5], the surgical team ensured that intravenous fluids and packed RBCs were readily available for prompt intervention if needed.

Postoperative pain management was a primary concern, as inadequate analgesia could worsen the patient’s restrictive lung disease by limiting effective ventilation and increasing the risk of respiratory complications. In this case, the use of a low, single dose of morphine carries a minimal transfer from mother to fetus, and transfer into colostrum is very low. The patient received a single dose of morphine in the immediate postoperative period, at which time breastfeeding had not yet been initiated, resulting in a very low risk of neonatal exposure. Although opioids were necessary, a multimodal approach was adopted to minimize their use and associated side effects. A combination of local anesthetic infiltration and nonsteroidal anti-inflammatory drugs was administered. In contrast, Dolak et al. [5] reported the use of a transversus abdominis plane (TAP) block as part of a multimodal strategy to reduce opioid requirements following cesarean delivery.

## Conclusions

This report highlights the importance of individualized anesthetic planning for patients with complex anatomical and physiological conditions. To our knowledge, this is the first reported use of CSA in a patient with JLS undergoing cesarean delivery. A multidisciplinary approach to address the unique challenges of JLS can help achieve safe perioperative and postoperative outcomes. By leveraging the expertise of multiple specialties and utilizing continuous spinal anesthesia to ensure adequate anesthetic control, this case demonstrates how careful planning and collaboration can optimize outcomes in high-risk obstetric surgery.
